# Cell shape determines gene expression: cardiomyocyte morphotypic transcriptomes

**DOI:** 10.1007/s00395-019-0765-7

**Published:** 2019-12-23

**Authors:** Payam Haftbaradaran Esfahani, Zaher ElBeck, Sven Sagasser, Xidan Li, Mohammad Bakhtiar Hossain, Husain Ahammad Talukdar, Rickard Sandberg, Ralph Knöll

**Affiliations:** 1 Department of Medicine, Integrated Cardio Metabolic Centre (ICMC), Heart and Vascular Theme, Karolinska Institutet, 141 57 Huddinge, Sweden; 2 Department of Cell and Molecular Biology (CMB), Karolinska Institutet, 171 77 Stockholm, Sweden; 3 Bioscience, Cardiovascular, Renal & Metabolism, BioPharmaceuticals R&D, AstraZeneca, 431 83 Gothenburg, Sweden

**Keywords:** Cell shape, Single-cell RNA sequencing, Gene expression and regulation, Myocardial biology

## Abstract

Cardiomyocytes undergo considerable changes in cell shape. These can be due to hemodynamic constraints, including changes in preload and afterload conditions, or to mutations in genes important for cardiac function. These changes instigate significant changes in cellular architecture and lead to the addition of sarcomeres, at the same time or at a later stage. However, it is currently unknown whether changes in cell shape on their own affect gene expression and the aim of this study was to fill that gap in our knowledge. We developed a single-cell morphotyping strategy, followed by single-cell RNA sequencing, to determine the effects of altered cell shape in gene expression. This enabled us to profile the transcriptomes of individual cardiomyocytes of defined geometrical morphotypes and characterize them as either normal or pathological conditions. We observed that deviations from normal cell shapes were associated with significant downregulation of gene expression and deactivation of specific pathways, like oxidative phosphorylation, protein kinase A, and cardiac beta-adrenergic signaling pathways. In addition, we observed that genes involved in apoptosis of cardiomyocytes and necrosis were upregulated in square-like pathological shapes. Mechano-sensory pathways, including integrin and Src kinase mediated signaling, appear to be involved in the regulation of shape-dependent gene expression. Our study demonstrates that cell shape per se affects the regulation of the transcriptome in cardiac myocytes, an effect with possible implications for cardiovascular disease.

**Electronic supplementary material:**

The online version of this article (10.1007/s00395-019-0765-7) contains supplementary material, which is available to authorized users.

## Introduction

Cardiomyocytes (CMs) generate, but also respond to, various types of biomechanical stress to maintain continuous contractile function of the heart. These hemodynamic constraints have profound effects on cellular architecture. They can either be short term, and last a matter of seconds, or they can be long term and last days, weeks, months, or even years. The long-term effects can include significant elongation of CMs, when sarcomeres are added in series, or thickening, when the sarcomeres are added in parallel [9]. Mutations affecting cardiac mechanotransduction are also involved and, by superimposition, these provide an additional level of complexity in the pathogenesis of cardiovascular diseases.

An important hemodynamic constraint is volume overload. The accompanying increased preload and enlargement of the ventricle leads to eccentric hypertrophy, with geometric changes in cellular shapes changing the aspect ratio (AR) for length and width from 7:1 (AR7) to about 11:1 (AR11). In addition, an increased afterload, namely a pressure overload, leads to concentric hypertrophy, which can, for example, change the cellular AR from 7:1 to 1:1 (AR1), and which can probably be observed in patients with hypertension [28] or other conditions [1, 10]. The consequences of these include the induction of specific sets of genes related to cardiac maladaptation, known as the fetal pattern of gene expression [17]. This type of expression is characterized by the downregulation of various genes, including beta receptors and sarcoplasmic reticulum ATPase (SERCA, Atp2a2) or isoforms, while activation of pro-apoptotic pathways is particularly prevalent in AR1 [9, 12]. However, these data have primarily been derived from whole heart gene expression studies, where it is difficult to identify the relative contribution of different cell types, and from in vitro studies that have analyzed bulk RNA. Moreover, there are a lack of transcriptome data on single cardiomyocytes (CM) [6, 8, 32] and the effect of cell shape on whole gene expression, namely mRNA transcriptome, has never been analyzed.

Mechanical forces are transmitted by integrins, which are heterodimeric, transmembrane receptors that are expressed in all cells, including those in the heart. They participate in multiple critical cellular processes including adhesion, extracellular matrix organization, signaling, survival, and proliferation. Integrins are particularly relevant for cardiomyocytes, as they translate mechanical into biochemical information [13].

Our interest in how cell shape may drive transcription [16] has been fueled by key studies that have demonstrated that similar types of cells with different curvatures show profound plasticity with regard to signaling, metabolic activity and differentiation [26]. In addition, Kuo et al. [21] showed that different shapes imposed on CMs had profound effects on functional parameters, such as calcium transients.

In summary, CMs are dynamic cells with the ability to change their shape during mechanically induced remodeling processes, and this affects their contractile functions and causes aberrations in cellular calcium metabolism [21]. This has led us to hypothesize that cell shape could have an impact on the transcriptome and that is why cell shape has been analyzed in our set of experiments.

## Materials and methods

**Fig. 1:**
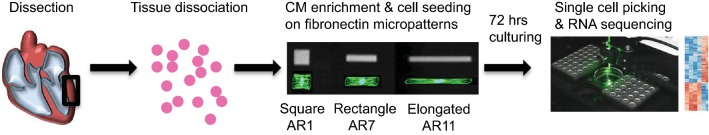
Schematic workflow of the single-cell experiment. Tissue was dissected from the left ventricle of neonatal rat hearts and dissociated into single cells. After enrichment, individual CMs were seeded on a chip containing fibronectin patterns with distinct ARs: AR1 (square), AR7 (rectangle), and AR11 (elongated). After a culturing period of 72 h, the whole chip was scanned and mononucleated single CMs that fully covered their patterns were selected. Selected cells were picked one by one using a semi-automated cell picker. Single-cell RNA-seq libraries were conducted following the Smart-Seq2 protocol

To investigate whether cell shape can alter the transcriptional plasticity of CMs, we cultured isolated single neonatal rat ventricular CMs (NRCM) on CYTOOchips (CYTOO, Cambridge, MA, USA) with fibronectin patterns of defined ARs. These included AR1, AR7, and AR11 (Fig. 1; Table 1 and Supplementary Fig. S1), which are referred to as morphotypes. AR7 was chosen to resemble the morphotypic dimensions of a normal CM in a healthy heart [2]. All cells were monitored by light microscopy and morphotypes were characterized using three-dimensional (3D) measurements of confocal image stacks (Supplementary Fig. S2). The chips were designed in a way that the surface areas of the different ARs remained constant, resulting in similar volumes (Table 1 and Supplementary Fig. S2b).

**Table 1:** Geometry and number of sequenced single cells

Morphotype	AR	Length (µm)	Width (µm)	Fibronectin area (µm^2^)	Number of cells analyzed/sequenced
AR1	1:1	47	47	2209	28/37
AR7	7:1	126	18	2268	31/41
AR11	11:1	155	14	2170	26/34
Unpatterned	–	–	–	–	24/32
Pre-patterned	–	–	–	–	27/36
Src-inhibited AR1	1:1	47	47	2209	14/19
Src-inhibited AR7	7:1	126	18	2268	14/20
Src-inhibited AR11	11:1	155	14	2170	14/17
β_1_-Integrin-inhibited AR1	1:1	47	14	2209	32/38
β_1_-Integrin-inhibited AR7	7:1	126	126	2268	33/40
β_1_-Integrin-inhibited AR11	11:1	155	155	2170	22/30
Src-overexpressed AR1	1:1	47	47	2209	23/29
Src-overexpressed AR7	7:1	126	126	2268	25/31
Src-overexpressed AR11	11:1	155	155	2170	26/31
Total					339/435

All the procedures involving animals were in accordance with the regulations of the animal ethics committee of the Karolinska Institutet, Stockholm, Sweden.

### Micropatterned chip

To grow CMs in specific rectangular shapes, we designed a micropatterned CYTOOchip: a squared glass coverslip with 3000 spatially distributed fibronectin micropatterns surrounded by a cytophobic surface. The chip was spatially divided into three zones and each zone resembled 1000 rectangular-shaped micropatterns, with specific AR pattern sizes of 11:1 (elongated), 7:1 (rectangle), and 1:1 (square), respectively, and a uniform surface area of 2200 µm^2^.

### Single-cell CM isolation

CMs were isolated and purified as previously described [24]. Hearts were rapidly excised from 2-day-old Sprague–Dawley rats and tissue samples were collected from the dorsal of the apex of the left ventricle. Single CMs were isolated from the tissue using the Neonatal Heart Dissociation Kit (Miltenyi Biotec, Bergisch Gladbach, Germany), combined with the gentleMACS Octo Dissociator (Miltenyi Biotec). Isolated cells were resuspended in DMEM:M199 (4:1), supplemented with 10% horse serum, 5% fetal bovine serum, and 1% penicillin/streptomycin (10,000 U/ml) (Thermo Fisher Scientific, Waltham, Massachusetts, USA). The suspension was pre-plated for 2 hours in 10 cm^2^ uncoated cell culture flasks (Nunc, Thermo Fisher Scientific) to remove the fibroblast and endothelial cells, by allowing non-CMs to attach to the surface of the culture flask. In addition, the non-adherent cells were magnetically labeled using a mixture of MicroBead-conjugated antibodies (Miltenyi Biotec) specific for non-CMs and removed by magnetic sorting. Enriched neonatal CMs were counted and diluted to a concentration of 3 × 10^4^ cells/ml. Four milliliters of the diluted CM suspension was dispensed over the CYTOOchips, sitting on a 35 mm Petri Dish, so that the cells attached to the fibronectin patches and acquired defined patterns. The chip remained submerged in media throughout the 72 h of culture. In addition, 4 ml CM suspension was cultured on fibronectin-coated 10 cm^2^ dishes to allow the cells to grow in unrestricted patterns. Both the patterned and unpatterned cells were treated with the same maintenance media throughout the 72-h culturing period. We also lysed some freshly isolated CMs immediately after isolation (pre-patterned cells).

### Cell volume measurement and 3D image reconstruction

After 48 h of plating, the patterned CMs were transfected with a CMV promotor-driven Cytosolic-YFP (pcDNA3.1(−)) reporter (Vector Biolabs, Malvern, PA, USA) to visualize the cytoplasm. After 72 h of aggregated culture time, the cells were fixed with 4% paraformaldehyde (PFA) in phosphate-buffered saline (PBS), washed three times with PBS, and mounted with Vectashield (Vector Biolabs) for confocal microscopy. Z-stacked images of the transfected cells were acquired using an A1R confocal microscope (Nikon, Tokyo, Japan), with a 63× oil-immersion objective and z-stack intervals of 100 nm. A 3D image was constructed and individual cell volumes were assessed using the volume measurement function of the NIS-Element image processing software (Nikon).

### Single-cell sorting and transcriptome library preparation

We used a semi-automated, micro-pipetting cell picker (CellSorter, Budapest, Hungary) to collect single cells from the chip submerged in culture [19]. After a culturing period of 72 h, the medium was replaced by 1:1000 Vibrant Dye Cycle green (Invitrogen, Carlsbad, CA, USA) in PBS-4:1 TryplE (Thermo Fisher Scientific), to visualize the nuclei of the live cells. We treated the cells with TryplE to loosen the cell attachment from fibronectin and to facilitate cell picking by fluidic vacuum. The entire chip was promptly scanned by a fluorescent microscope connected to the cell picker, before the cells became rounded due to TryplE treatment. We only selected those micropatterns that contained a mononucleated single cell and only when the cell fully covered its fibronectin micropattern. The XY coordinate positions of the selected cells were recorded by the cell picker software for subsequent picking. The selected cells were picked one by one using a microcapillary pipette, which traversed to the recorded coordinate of each selected cell and applied a pre-defined level of vacuum to pick that particular cell. Only a fraction of the selected cells (about one-third) were picked, because either the cell was too tightly attached to the fibronectin patch and vacuum pressure was not strong enough to pick up the cell without damaging it or the cell became too loose due to the TryplE treatment and got detached by the approaching needle before being picked. The successfully picked single cells were lysed immediately in individual polymerase chain reaction (PCR) tubes containing 2 μl of Smart-Seq2 cell lysis buffer [30]. The entire process, which started with removing the media, was completed within 30 min. The completely lysed single cells were stored at − 80 °C overnight before complementary deoxyribonucleic acid (cDNA) synthesis, using the Smart-Seq2 protocol [30]. The quality of the cDNA was checked using Agilent Bioanalyzer (Agilent, Santa Clara, CA, USA) and RNA-Seq libraries were prepared using in-house compatible Tn5 and Nextera index primers (Illumina, San Diego, CA, USA). After a final clean-up, the size distribution of the sequence libraries was checked using Agilent high-sensitivity chip and the concentration of each library was measured using the Qubit 3 Fluorometer (Invitrogen).

### Single-cell mRNA sequencing and gene expression analysis

Single-cell RNA sequencing was performed at the sequencing facility at the Karolinska Institutet using the Genome Analyzer HiSeq2500 (Illumina) for single-end sequencing of 56 bps. We sequenced a total of 213 single cells (Table 1). The Genome Analyzer Analysis Pipeline (Illumina) was used to process the sequence files of raw reads in the FASTQ format. All sequences were deposited in the Sequence Read Archive at http://www.ncbi.nlm.nih.gov/sra. The sequence reads were aligned to the RGSC Rnor 6.0 using Tophat2, combined with Bowtie2 [15, 22]. FeatureCounts software was used to count the mapped reads for each gene and uniquely mapped reads were considered as counts and analyzed further [23]. We performed quality control analysis for individual cells, based on the total number of counts (Supplementary Fig. S3) and the proportion of spike-in transcripts. Cellular cardiac identity of the single cells was confirmed by the expression of known CM-specific transcript markers, such as Tnnt2, Tnni3, and Myh6. A total of 178 cells out of 213 passed the quality control assessment (Table 1). The genes with average counts of > 1 across all cells were selected for further analysis. Normalization was performed based on external RNA control consortium (ERCC) spike-in coverage. Since the same amount of ERCC spike-in RNA was added to each sample, any differences in the coverage of ERCC spike-in RNA were due to technical noise and removed. Therefore, the size factor for each cell was defined as the sum of all the spike-in counts in each cell. For normalization, size factors were scaled, so that the average of all size factors was unity. Normalized expression values were computed by dividing the counts for each cell by the size factor for that cell. The computeSpikeFactors function from the scran Bioconductor package was used to calculate specific size factors for ERCC spike-ins. Then, the expression values of the endogenous genes were normalized by equalizing the total ERCC spike-in counts across the cells using the normalize function of the scater package [25]. Log-normalized values were calculated by adding one to the normalized count and performing a log2 transformation. The Seurat package [4] in R (The R Foundation, Vienna, Austria) was applied to perform differential gene expression analysis between different morphotypes. The differentially expressed genes (DEGs) were identified based on adjusted *p* < 0.05 for multiple-testing correction, using the Benjamini–Hochberg method.

### Ingenuity pathway analysis (IPA)

The significant DEGs were identified in pairwise comparisons of morphotypes (i.e., AR1 versus AR7, AR11 versus AR7, and AR1 versus AR11) and analyzed using IPA software (QIAGEN, Hilden, Germany) to identify enriched canonical pathways and diseases and biological functions [20]. Three sets of DEGs were imported to the IPA software, along with their regulation directions (up or down) and magnitudes (log2 fold change). The right-tailed Fisher’s exact test was used to calculate the gene set enrichment *p* values associated with a given canonical pathway or biological function. The enrichment *p* values indicated whether it was likely that the similarity between the set of DEGs and a specified canonical pathway or biological function was random [20]. The enrichment *p* value was then adjusted using the Benjamini–Hochberg method for multiple-testing and false discovery control. Furthermore, the regulatory effect of the interactions between the DEGs was measured by the bias-corrected activation z-score, with regard to the regulation patterns of the genes [20]. The enriched canonical pathways were reported according to their −log (Benjamini–Hochberg *p* value) and heatmapped showing the predicted level of activation (red) or inhibition (blue). The impact of DEGs on diseases and biological functions was determined by calculating the bias-corrected z-score.

### Fluorescent immunostaining

After 72 h in culture, NRCMs were incubated for 30 min at 37 °C with the MitoTracker Deep Red probe (Cell Signalling Technology, Danvers, MA, USA) and diluted in growth media to a final concentration of 500 nM. The thickness of our CYTOOchip was not adequate for confocal imaging, and we had to fix the cells and use an inverted confocal microscope. After incubation, the cells were briefly rinsed in warm PBS and fixed in 4% buffered PFA for 10 min at room temperature and rinsed three times with ice-cold PBS for 5 min. The cells were permeabilized in 0.2% Triton X-100 (Sigma-Aldrich, St. Louis, MO, USA) in PBS for 10 min and blocked in 1% bovine serum albumin and glycine (22.52 mg/ml) in PBST (PBS + 0.1% Tween 20) for 30 min.

In addition, NRCMs were stained for sarcomeric α-actinin clone EA-53 (Sigma-Aldrich) by incubating the cells at 4 °C overnight. The cells were incubated with Donkey Anti-Mouse IgG Alexa Fluor 488 ab150105 1:800 (abcam, Cambridge, United Kingdom) for 1 h at room temperature for the secondary staining and chromatin was stained by DAPI (Molecular Probes, Eugene, OR, USA). Fluorescence imaging was performed with an inverted SP8 confocal microscope (Leica, Wetzlar, Germany), using a 63× oil-immersion objective.

### β1-integrin inhibition

We inhibited β1-integrin in three different patterned CMs. CMs were cultured in CYTOOchips for 54 h and were incubated with anti-integrin-β1 specific antibody (10 µg/ml, cat. no. 555002) (BDTransduction Laboratories, San Jose, CA, USA) for 18 h prior to picking. During this 18 h of antibody incubation, patterned CMs were field-stimulated to contract using C-Pacer (IonOptix, MA, USA) at 0.5 Hz. Single cells were then picked after 72 h of total culturing (18 h of inhibition) and sequenced as described for single-cell sequencing. We only picked cells that fulfilled the study criteria, namely single cells with a fibronectin micropattern.

### Src kinase inhibition

We inhibited Src kinase in three different patterned CMs. CMs were cultured in CYTOOchips for 24 h, before adding saracatinib (AstraZeneca, Cambridge, UK) [11] at a concentration of 1 μM. Single cells were picked after 72 h of total culturing (48 h of inhibition) and sequenced as described for single-cell sequencing. We only picked cells that fulfilled the study criteria, namely single cells with a fibronectin micropattern.

### Src kinase overexpression

We overexpressed Src in three different patterned CMs. CMs were cultured in CYTOOchips for 24 h, before being transduced by 4 × 10^6^ pfu/ml Adenovirus-Kras-Src (Vector Biolabs) [34] for 48 h. During the last 18 h of adenovirus transduction, patterned CMs were field-stimulated to contract using C-Pacer (IonOptix) at 0.5 Hz before being picked. Single cells were picked after 72 h of total culturing (48 h of transduction) and sequenced as described for single-cell sequencing. We only picked cells that fulfilled the study criteria, namely single cells with a fibronectin micropattern.

### Statistical analyses

We used GraphPad Prism version 7.00 for Windows (GraphPad Software, CA, USA) for the statistical analysis, so that we could compare different morphotypes of gene expression. Gene expression data are presented as boxplots, with boxes representing median, 25% lower and 75% upper percentiles, and whiskers representing the minimum and maximum values. Comparisons between the different morphotypes were performed using the unpaired Student’s *t* test. We performed multiple-testing corrections for differential gene expression analysis. The statistically significant DEGs were used for further analysis using IPA software. Unsupervised clustering using three-dimensional t-distributed stochastic neighbor embedding (t-SNE) and principal component analysis (PCA) were performed in R version 3.5.0 (The R Foundation). Venn diagrams identifying common DEGs were drawn using R. An adjusted *p* value of less than 5% was considered statistically significant.

## Results

### Lower number of detected genes in pathological ARs

To evaluate possible effects of cell shape on gene expression, we isolated single NRCMs and patterned them on CYTOOchips with fibronectin patches of defined ARs, namely morphotypes AR1, AR7, and AR11 (Fig. 1; Table 1 and Supplementary Fig. S1). In accordance with previously published reports, CMs with shapes corresponding to AR7 were chosen to resemble the morphotypic dimensions of normal CMs in a healthy heart [2]. All cells were monitored by light microscopy and morphotypes were characterized using 3D measurements of confocal image stacks (Supplementary Fig. S2). The chips were designed, so that the surface areas of the different ARs remained the same and this resulted in similar volumes (Table 1 and Supplementary Fig. S2b).

Similar to Kuo et al. [21], cells were harvested after 72 h of plating. Only those single cells that fully covered the fibronectin islands of micropatterns were selected using software (CellSorter) for picking, and immediately lysed in individual PCR tubes containing 2 μl of Smart-Seq2 cell lysis buffer [30]. The quality of the cDNA was checked using the Agilent Bioanalyzer (Agilent) and RNA-Seq libraries were prepared using in-house compatible Tn5 and Nextera index primers (Illumina). After a final clean-up, the size distribution of the sequence libraries was checked using the Agilent high-sensitivity chip and the concentration of each library was measured using the Qubit 3 Fluorometer (Invitrogen).

Single-cell RNA sequencing was performed at the in-house sequencing facility at the Karolinska Institutet using the Genome Analyzer HiSeq2500 (Illumina) for single-end sequencing of 56 bps.

**Fig. 2:**
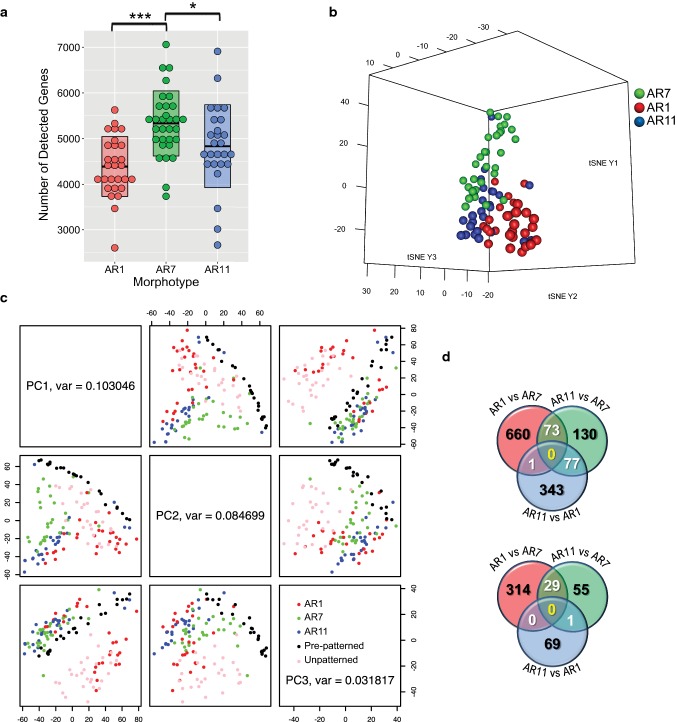
Transcriptomic profiles of single CMs in three different ARs: AR1 (square), AR7 (rectangle), and AR11 (elongated). **a** Boxplot of the number of genes detected in three different morphotypes. The box represents the median, 25% lower and 75% upper percentiles, and the whiskers show the ranges. *p < 0.05, ***p < 0.001 (unpaired Student *t* test). **b** Three-dimensional t-SNE projection of 85 patterned CMs of different morphotypes (AR7, *n* = 31; AR1, *n* = 28; AR11, *n* = 26), based on all expressed genes. Different morphotypes are color-coded according to ARs. **c** PCA plots of 85 patterned, 24 unpatterned and 27 pre-patterned CMs, based on whole-genome expression values. Three principal components corresponding to three largest eigenvalues are shown. **d** Venn diagrams showing the number of significantly (false discovery rate-adjusted *p* value < 0.05) downregulated (top panel) and upregulated (bottom panel) genes in different morphotypic comparisons

We sequenced a total of 213 single cells with different morphotypes (Table 1) and detected an average of 4852 genes per cell (range 2603–7060). A gene with a read per kilobase million (RPKM) value of > 1 was considered as detected (Fig. 2a). The average number of detected genes in AR7 was 5332 and the number of detected genes was significantly lower in both AR1 (4391, *p* < 0.001) and AR11 (4835, *p* < 0.05), when compared to AR7.

### Gene expression heterogeneity due to different cell shapes

The next step was to investigate whether cell shapes per se drove gene expression heterogeneity. We did this by performing unsupervised clustering based on all the expressed genes and we observed that the cells with similar morphotypes clustered together in three-dimensional t-SNE plots (Fig. 2b). In line with this, and to find out to what extent our morphotypes possibly changed the transcriptome, we compared patterned cells with unpatterned and pre-patterned CMs. The unpatterned cells were cultured cells that were allowed to take any shape, as detailed in the Methods section, and the pre-patterned cells were those where the cells lysed immediately after isolation. PCA plots were constructed based on the log-normalized expression values of all the expressed genes (Fig. 2c). Patterned cells tended to cluster together, depending on their ARs, but unpatterned and pre-patterned cells were more diverse, suggesting that arbitrary cellular shapes lead to diverse gene expression. Although the unpatterned cells showed more divergence, they partially overlapped the patterned cells, which probably reflected the same length of cell culture. However, the pre-patterned cells, which were never cultured, were even more diverse and distant from the other patterned and unpatterned cells (Fig. 2c). When we compared PC1 and PC3, we observed that the pre-patterned cells overlapped with the different patterned cells.

### Downregulation of genes in pathological morphotypes

To analyze the consequences of the morphotypic heterogeneity in greater detail, we identified significant DEGs by comparing different morphotypes and presenting them as a Venn diagrams (Fig. 2d). The complete list of DEGs is provided in Supplementary Table S1. We observed that 734 and 280 genes were significantly downregulated in AR1 and AR11, respectively, compared to AR7. In addition, AR1 and AR11 shared 73 genes in common. On the other hand, only 343 and 85 genes were upregulated in AR1 and AR11, respectively, compared to AR7, with 29 common genes (Fig. 2d). These results suggest that fewer genes were upregulated in pathologic morphotypes. Together with the overall loss of gene expression observed in AR1 and AR11, these data support the notion that elongation of CMs or squaring of CMs had powerful effects on the number of expressed genes. Moreover, the differential gene expression analysis was performed by comparing AR7 versus unpatterned and pre-patterned cells. The complete list of DEGs can be found in Supplementary Table S2.

### Canonical pathways and biological processes were influenced by different cell shapes

**Fig. 3:**
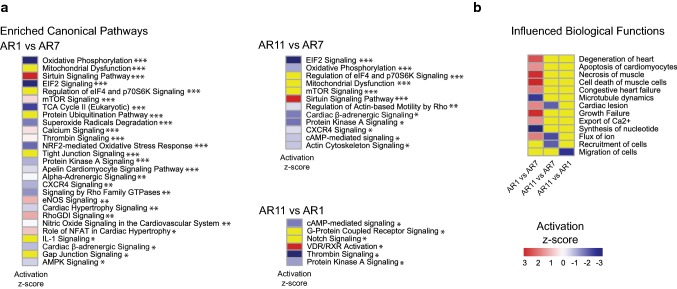
Canonical pathways and various cellular processes influenced by different ARs. **a** The activation z-scores of the enriched pathways for different pairwise morphotypic comparisons (AR11 versus AR7, AR11 versus AR1, and AR1 versus AR7) are represented by heatmaps. Blue = inhibited, red = activated and yellow = no suggested value for activation z-score by IPA. *p_*BH*_ < 0.2, **p_*BH*_ < 0.1, ***p_*BH*_ < 0.05. **b** The z-score (bias-corrected) heatmap of biological functions and cellular processes influenced by DEGs in three pairwise morphotypic comparisons. Blue = inhibited, red = activated, and yellow = no suggested value for activation z-score by IPA

While the analysis of a single DEG can provide important information, this analysis needs to be complemented by unsupervised bioinformatic approaches. In this study, we applied pairwise comparisons of morphotypes (i.e., AR1 versus AR7, AR11 versus AR7, and AR1 versus AR11) using IPA software [20]. This enabled us to identify a variety of enriched canonical pathways, as well as diseases and biological functions (Fig. 3). Oxidative phosphorylation, eukaryotic initiation factor 2, protein kinase A, and cardiac beta-adrenergic signaling were inhibited in both AR1 and AR11, when compared to AR7, suggesting that common pathways are affected in pathological conditions. On the other hand, Sirtuin signaling was activated in both AR1 and AR11 compared to AR7 (Fig. 3a). We observed upregulation of vitamin D receptor/retinoid X receptor (VDR/RXR) activation and downregulation of cyclic adenosine monophosphate-(cAMP) mediated signaling, in AR11 compared to AR1 (Fig. 3a). We also investigated the cellular processes that are affected by DEGs due to morphotypic variations. Degeneration of the heart, apoptosis of CMs, and necrosis of muscle were predicted to be activated in AR1 versus AR7. However, we observed lower flux of ion and recruitment of cells in AR11 versus AR7 and less migration of cells in AR11 versus AR1 (Fig. 3b). When we took these differences into account, it was interesting to note that changes in cell shape not only caused changes in function [21], but they also caused profound alterations in gene expression.

### Sarcomeric and mitochondrial structure of patterned NRCMs

**Fig. 4:**
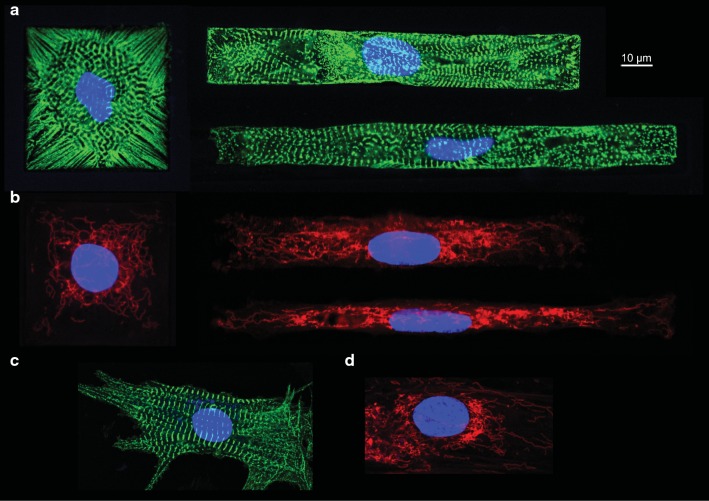
Structural aspects of various morphotypes. **a** α-actinin sarcomeric structures (green) and DNA (blue) of the patterned CMs with different ARs. Changes in the orientation of sarcomeres are observed in AR1. **b** Mitochondrial staining (deep red) of patterned CMs with various ARs. Mitochondria have been stained using MitoTracker Deep Red. **c** α-actinin sarcomeric structures (green) and DNA (blue) of an unpatterned CM. **d** Mitochondrial staining (deep red) of an unpatterned CM

Structural aspects of patterned CMs were examined by staining sarcomeric structures using an alpha-actinin antibody (Fig. 4a). Based on this analysis, we did not observe overall changes in sarcomere structure, but we did observe changes in the orientation of sarcomeres, which appeared more radial in AR1.

Taking into account the fact that mitochondrial pathways were significantly affected, we used the MitoTracker (Cell Signalling Technology) to visualize mitochondria in patterned NRCMs with various ARs (Fig. 4b). We did not observe major mitochondrial network changes: sarcomeric and mitochondrial staining of an unpatterned CM is also shown in Fig. 4c, d. An in-depth analysis of the effects of cell shape on overall cell structure and mitochondrial function is in progress, but that is probably beyond the scope of this paper.

### Effects of mechanotransduction pathways on the functional consequences of various ARs

A wide variety of different signal transduction cascades are involved in cardiac mechanotransduction, including nuclear lamina proteins, ion channels, mitogen-activated protein kinases, membrane receptors such as ATR1, and integrins that include Src kinase [5]. As the integrin/Src kinase pathway is probably one of the best-known factors involved in mechanotransduction, but not the only one, we determined the effects of this pathway first [32].

**Fig. 5:**
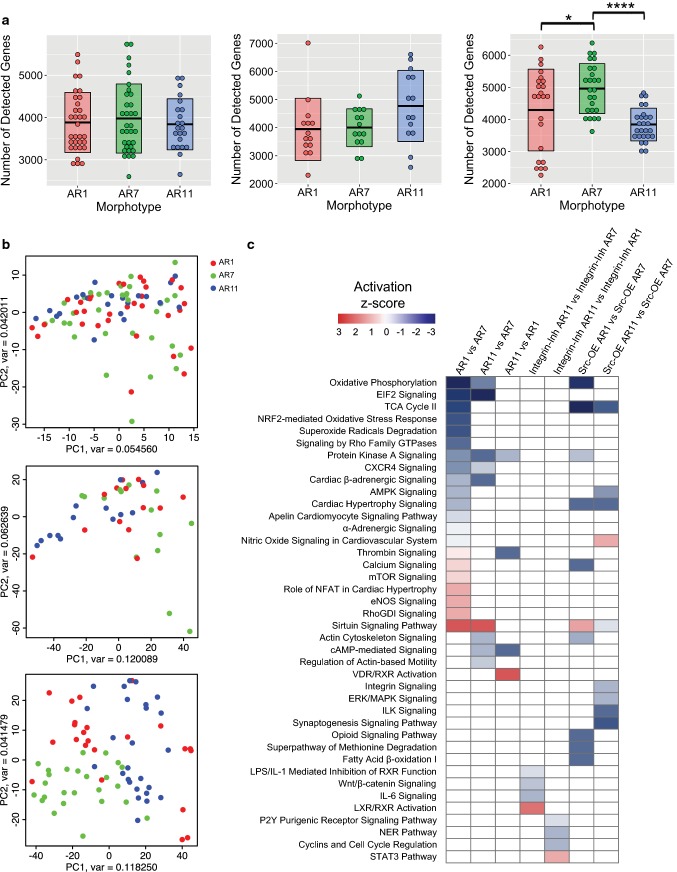
Mechanotransduction functional studies. **a** Boxplots presenting number of detected genes per morphotypes in β1-integrin-inhibited treatment (left panel), Src-inhibited treatment (middle panel), and Src-overexpressed treatment (right panel). **b** PCA plots based on all expressed genes for β_1_-integrin-inhibited treatment (top panel), Src-inhibited treatment (middle panel), and Src-overexpressed treatment (bottom panel). **c** The activation z-scores of the enriched IPA canonical pathways for different pairwise morphotypic comparisons are represented by heatmaps. Blue = inhibited and red = activated. Src-inhibited treatment is not presented, since no pathway is significantly enriched in Src-inhibited morphotypic comparisons

We started by inhibiting β_1_-integrin in all three morphotypes with blocking anti-β_1_-integrin antibody (10 µg/ml, cat. no. 555002) (BD Transduction Laboratories). After quality control, we analyzed a total of 87 sequenced β_1_-integrin-inhibited single cells with different morphotypes (Table 1) and detected an average of 3908 genes per cell (range 2596–5754). The average number of detected genes was 3884, 3978, and 3840 in β_1_-integrin-inhibited AR1, AR7, and AR11 morphotypes, respectively. Moreover, the number of detected genes was not significantly different between morphotypes of β_1_-integrin-inhibited condition (Fig. 5a, left panel).

We inhibited Src with saracatinib, a known Src inhibitor [11], in all three morphotypes and sequenced 14 Src-inhibited single cells from each AR, making a total of 42 cells. We performed intra-condition studies, by comparing the number of genes detected between treated morphotypes in each condition. A gene with a read per kilobase million (RPKM) value of ≥ 1 was considered as detected. The average of 4235 genes per cell (range 2294–7012) was detected among all Src-inhibited single cells, while the average number of 3938, 3996, and 4771 was detected, respectively, in Src-inhibited AR1, AR7, and AR11 morphotypes. Moreover, the number of detected genes was not significantly different between all Src-inhibited morphotypes (Fig. 5a, middle panel). In a third, related set of experiments, we overexpressed Src by transducing patterned CMs with the adenovirus–Kras–Src vector in all three morphotypes [34]. After quality control, we analyzed a total of 74 sequenced Src-overexpressed single cells with different morphotypes (Table 1) and detected an average of 4360 genes per cell (range 2251–6382). The average number of detected genes in Src-overexpressed AR7 was 4963 and the number of detected genes was significantly lower in both Src-overexpressed AR1 (4293, *p* < 0.05) and Src-overexpressed AR11 (3838, *p* < 0.0001), when compared to Src-overexpressed AR7 (Fig. 5a, right panel).

Next, we investigated the heterogeneity in whole-genome expression profiles between morphotypes of each treated condition. We constructed intra-condition PCA plots, using all expressed genes in the samples of each condition (Fig. 5b). Importantly, we observed that Src-overexpressed morphotypes clustered according to their morphotypes (Fig. 5b, bottom panel), whereas β_1_-integrin-inhibited and Src-inhibited morphotypes were intermingled (Fig. 5b, top and middle panels). This indicates that inhibition of the integrin/Src kinase pathway at different levels significantly interfered with shape-dependent effects on gene expression and hence links mechano-sensation to regulation of the transcriptome.

To further explore the role of integrin-mediated and Src-mediated mechanosensing pathways on the outcomes of various cell shapes, we identified DEGs, comparing the intra-condition morphotypes (Supplementary Tables S3–5). We derived the enriched IPA canonical pathways and biological functions when we pairwisely compared morphotypes within each treated condition (Fig. 5c).

Interestingly, no canonical pathway or biological function was significantly enriched in any pairwise comparison of Src-inhibited morphotypes.

These results emphasize that inhibition of β_1_-integrin and of Src abate the effects of cell shape in terms of number of detected genes, heterogeneity in expression profiles, and enriched canonical pathways.

On the other hand, intra-condition comparison of Src-overexpressed morphotypes showed that the number of detected genes was significantly lower in AR1 and AR11 compared to AR7, an effect which was seen in intra-condition comparisons of untreated condition. Moreover, a distinct heterogeneity between expression profiles of different Src-overexpressed morphotypes was revealed in PCA plots, while similar feature was represented in the gene expression PCA plot of untreated morphotypes. Furthermore, cAMP-mediated signaling, protein kinase A signaling, and sirtuin signaling pathway were enriched when we compared AR11 vs AR7 morphotypes in both Src-overexpressed and untreated conditions. Likewise, cardiac hypertrophy signaling, protein kinase A signaling, calcium signaling, sirtuin signaling, and oxidative phosphorylation canonical pathway were enriched, comparing AR1 vs AR7 morphotypes in both Src-overexpressed and untreated conditions.

To evaluate the features of the DEGs, we additionally performed Gene Ontology (GO) enrichment analysis, using PANTHER classification system. Significantly enriched GO biological processes were identified using the Fisher’s exact test type, corrected by false discovery rate. Particularly, “regulation of signaling”, “cellular response to chemical stress”, “regulation of signal transduction”, “response to external stimulus”, and “response to stress” were among significantly enriched GO terms when we compared β_1_-integrin-inhibited AR11 vs β_1_-integrin-inhibited AR7. Furthermore, the “cell surface receptor signaling pathway” was enriched when we compared β_1_-integrin-inhibited AR7 with both β_1_-integrin-inhibited AR1 and AR11 (Supplementary Fig. S4).

On the other hand, “cardiac muscle hypertrophy in response to stress”, “regulation of cardiac muscle cell contraction”, “sarcomere organization”, and “regulation of the force of heart contraction” were significantly enriched when we compared Src-overexpressed AR7 with both Src-overexpressed AR1 and AR11 (Supplementary Fig. S5). Src-inhibited treatment is not presented, since no GO term was significantly enriched in Src-inhibited morphotypic comparisons.

These findings suggest possible effects of mechanotransduction on morphotype-dependent gene expression via the integrin/Src kinase system as inhibition of this pathway abates features of morphotype-dependent gene expression, whereas overexpression of Src pronounced similar attributes of cell shape.

## Discussion

Different types of cardiac hypertrophy at the organ level are associated with increased CM volumes, or cellular hypertrophy, and consecutive changes in cell shape. However, these are not the only associations. While the effects of cell volume (i.e., cardiac hypertrophy) on gene expression are relatively well known and have been studied for many years [9], the effects of cell shape on gene expression are not so well understood. Therefore, this study was designed to systematically analyze the effect of cell shape on the mRNA transcriptome. To do this, we developed a novel single-cell patterning strategy, followed by single-cell RNA sequencing. This allowed us to profile the transcriptomes of single CMs of defined geometrical morphotypes and characterize these according to a range of physiological or pathological conditions, namely afterload/concentric versus preload/eccentric.

We detected an average of 4852 genes in different morphotypes, which was well within the range of genes expressed in other cell types, such as endocrine cells [33]. However, the number of genes detected in pathological morphotypes (AR1 and AR11) was significantly lower than those in AR7. The Venn diagrams (Fig. 2d) also show that the pathological morphotypes demonstrated overall downregulation of gene expression. This observation may have important implications for different types of cardiac conditions, where CMs undergo characteristic changes in cell shape and where silencing of important genes may occur. Of the 1077 DEGs that were identified when we compared AR1 and AR7, 734 were downregulated in AR1. Of the 365 DEGs that where identified when we compared AR11 and AR7, 280 were downregulated in AR11. This provides additional support for the hypothesis that changes in AR primarily lead to the downregulation of global gene expression. When we analyzed the list of DEGs in terms of biological processes (AR1 versus AR7, AR11 versus AR7, and AR11 versus AR1), this revealed that the overwhelming number of affected cell functions included translation, biological processes, and signal transduction. However, transcription factors did not appear to play a major role, at least not in quantitative measures. This was probably not very surprising, as the changes in gene expression were probably primarily due to changes in cell shape, which indicates that membrane-related signaling was dominant, including integrin signaling, ion transport, signal transduction, and cell–cell communication.

However, the most important observation to emerge from our study was that the three different morphotypic transcriptomes demonstrated significantly different gene expression profiles. In other words, cell shape determined gene expression. This was evident from the different clusters that we observed in unsupervised clustering in the t-SNE and PCA analysis, which was based on all of the expressed genes. While hypertrophy is relatively well known to cause profound changes in gene expression [18], to the best of our knowledge, no studies have ever reported cell shape-dependent changes in single cells covering whole mRNA transcriptomes.

Analysis of the DEGs identified a number of genes known to be involved in cell survival or cell death. These were genes affecting calcium metabolism (such as S100A 4 and SERCA2a-Atp2a2, which are significantly lower in AR1 versus AR7), mitochondrial energy metabolism (namely Atp5e, Atp5mf, and Atp5mg, which are significantly lower in AR1 and AR11 versus AR7), and protein metabolism (multiple ribosomal proteins, which are significantly down in AR11 versus AR7). Based on these data, we started to analyze possible structural effects due to differences in ARs by staining sarcomeric structures using an alpha-actinin antibody (Fig. 4a). Overall, we did not observe changes in sarcomere structure, but we noticed radial orientation of sarcomeres in AR1.

In addition, we used MitoTracker to visualize mitochondria in patterned NRCMs with various ARs. Although some genes essential for mitochondrial function were significantly changed in AR1 and AR11, we did not observe any overt structural defects in mitochondria (Fig. 4b). An in-depth analysis of the effects of cell shape on overall cell structure and mitochondrial function is in progress, but probably beyond the scope of this manuscript.

**Fig. 6:**
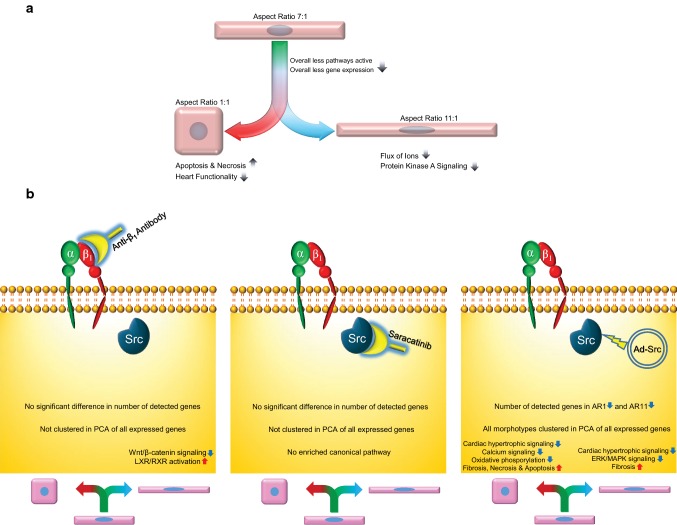
Summary figure. **a** Gene expressions and pathways are suppressed in both untreated AR1 and AR11 morphotypes. Important biological functions affected by the suppressed gene expressions are shown. **b** Important outcomes of β1-integrin-inhibited treatment (left panel), Src-inhibited treatment (middle panel), and Src-overexpressed treatment (right panel) on different morphotypes. Please note the almost identical reaction of cells in response to β1-integrin and Src kinase inhibition. Inhibition of this pathway abolishes shape-dependent gene expression, while Src kinase activation preserves this effect. These both suggest an important role of this pathway in morphotype-specific control of the mRNA transcriptome

In line with the significant downregulation of gene expression in pathological morphotypes, IPA predicted that the majority of canonical pathways also downregulated in AR1 and AR11 (Figs. 3a, 6a).

Indeed, IPA analysis identified a variety of pathways that were compatible with general features of cardiac maladaptation, especially the upregulation of Nppa, Nppb, Actc1, Slc8a1 (sodium calcium exchanger), calcineurin (Ppp3ca), and Rcan1 in AR11 [17]. The significant downregulation of Atp2a2 (SERCA2a) in AR1 is of particular interest, as this will unequivocally contribute to defects in calcium transients [27]. However, another important finding of this study was the activation of pro-apoptotic, pro-cell death-related pathways in AR1 versus AR7 (biological functions, Fig. 3b), predicted by IPA.

Direct analysis of the pathways active in AR11 versus AR1 identified that only VDR/RXR signaling was activated, while cAMP-mediated signaling, thrombin signaling, and protein kinase A signaling were less active. Activation of VDR/RXR signaling, which is partially involved in the activation of extracellular signal-regulated kinases 1/2/mitogen-activated protein kinase 1/2, protein kinase C, and protein kinase A signaling cascades, can probably be seen as protective in this context as it counteracts the loss of beta-adrenergic signaling.

Previous studies have shown that transgenic overexpression of Ras [35] and mitogen-activated protein kinase 1 [3] in CMs in vivo activated extracellular signal-regulated kinases 1/2 and resulted in concentric hypertrophy, thus forcing CM into AR1-like shapes. Members of this pathway can also lead to the activation of calcineurin/nuclear factor of activated T-cell cytoplasmic, a well-known driver of cardiac hypertrophy [9], which is significantly induced in AR1 (Fig. 3a).

Protein kinase A/cAMP signaling can be regarded as mechanosensitive and membrane tension dependent [29] and was found different in AR1 and AR11 in our experiments (Fig. 3a). Taking into account the fact that the membrane surface was lowest in AR1, and, therefore, the membrane tension was highest, inhibition of this pathway provides a molecular link between cell shape and gene expression. This may lead to extracellular signal-regulated kinases 1/2 activation. This is known to play a role in concentric hypertrophy [9], which also points to the induction of self-regulatory pathways.

Apart from that, a variety of disease-related and toxicological pathways were affected in pathological morphotypes, particularly in AR1. These included prominent pathological functions, such as degeneration of the heart, apoptosis, cell death, and microtubule dynamics (Fig. 3b).

It is remarkable to see that defined geometrical morphotypes that are characteristic of pathological conditions mimic the activation of pathways observed in many types of cardiac maladaptation, at least to some extent. These include the loss of beta-adrenergic signaling, mainly in AR11, defective oxidative phosphorylation in both pathological shapes and less SERCA activity, which was demonstrated in our experiments by a significant loss of Atp2a2, in AR1. We also observed increased expression of atrial natriuretic factor (Nppa) in AR11, but not in AR1, well known to be activated when the CM is stretched [7].

Moreover, we found significant changes in membrane receptors for growth hormones and signaling. This raises questions about what their function would be with regard to CM behavior if the receptor ligands were applied, for example G protein-coupled receptors 157 and 179 (Supplementary Table S1). Intriguingly, we did find that there were generally different responses for hypertrophic markers in AR11 than AR1 morphotypes and this indicates different strategies for cellular responses, depending on the lateral or radial polarity of the cell. Clearly, regulatory pathways that are not similar to each other are activated by different shapes (Fig. 3a, c). To the best of our knowledge, our study is the first one to demonstrate the fact that cell shape has profound effects on the mRNA transcriptome of single cells.

The effects of β_1_-integrin and Src kinase inhibition, whereby shape-dependent effects are abrogated, point to the fact that this pathway plays an important role in shape-dependent effects on gene expression, with the strongest effects being seen for AR11. The effects on AR11 were expected, as AR11 is predicted to have significantly more costameres and, therefore, more Src kinase action points than AR1 or AR7. Overexpression of Src kinase by means of an adenovirus has the opposite effect, by keeping in place the significantly lower number of gene expressed in AR1 and AR11 compared to AR7, an effect seen in untreated cardiomyocytes. Apart from that, a distinct heterogeneity between expression profiles of different Src-overexpressed morphotypes was revealed in PCA plots, similar to what was found in untreated morphotypes (Fig. 6b).

This study yielded information on some of the first CM-specific transcriptomes, which will provide a valuable resource for researchers in the field [6, 8, 32]. In addition, we mimicked in vitro characteristic shapes imposed on CM in vivo by genetic or hemodynamic constraints and identified the tight interplay between cellular architecture and gene expression. This relationship was characterized by the fact that deviation from the normal cell shape was associated with deactivation of specific canonical pathways and significant downregulation of gene expression in pathological cell shapes. Both of these were followed by the induction of maladaptive pathways. Moreover, qualitative imbalances were observed between pathologic shapes, particularly with regards to pathways involving cell survival or cell death, beta-adrenergic signaling, energy metabolism, and calcium metabolism (Figs. 3b, 6a).

Last but not least, we wish also to refer to some of the limitations of our study. We only used neonatal CMs to generate different morphotypes, as the adult CMs are notoriously difficult to culture and maintain in a specific shape. Furthermore, we cultured the CMs for 72 h ex vivo, which might have affected the overall gene expression pattern. However, this culturing was necessary for the cells to form specific morphotypes. We used Smart-Seq2 protocol for RNA sequencing, which only targets poly-A tail containing mRNAs and only about 50% of the detected reads were uniquely mapped. Finally, we only selected those single cells that were mononucleated and fully covered the fibronectin micropattern. These strict criteria, and difficulties in proper loosening of the adherent cells for successful picking, allowed us to have limited number of cells from each morphotype.

Nevertheless, the signaling dynamics that are involved in the process cell shape-dependent gene expression appear to serve as a positive feedback control mechanism, which dynamically regulates gene activity [31]. This process is thought to be bidirectional, as gene expression patterns are tuned due to shifted cell shapes.

This paper reports the development of a novel platform for studying cardiomyocytes and possibly cardiac disease in vitro in future, similar to what has recently been achieved for liver (i.e. “liver on a chip”, [14]) and identifies cell shape as a powerful determinant of gene expression in general, as it affects the transcriptome as a whole, at least the mRNA transcriptome. These findings potentially represent an important and novel observation, with far-reaching implications for biology and medicine. The American Food and Drug Administration and the pharmaceutical industry have introduced mandatory preclinical drug screening guidelines to detect potential cardiac toxicity before any drugs are released onto the market. Based on our data, the CMs used in these tests should have physiological shapes.

## Electronic supplementary material

Below is the link to the electronic supplementary material.

Supplementary material 1 (EPS 80073 kb) Layout of the chip with fibronectin micropatterns. **a** Image of a CYTOOchip. The chip is a 19.5×19.5 mm coverslip with fibronectin micropatterns, printed by photolithography on a borosilicate glass. **b** Chip layout. The chip is divided to three zones and each zone consists of fibronectin micropatterns with specific AR. Fluorescent images of different shapes of fibronectin micropatterns are shown in magnified view for each zone

Supplementary material 2 (EPS 2854 kb) Volume measurements of single cells with different ARs. **a** AR7 (top), AR11 (middle) and AR1 (bottom). The 3D images have been reconstructed by 3D registration of 100 z-stack images. Cells were transfected by Cyto-Src-YPet-reporter (Vector BioLabs) and then fixed. Z-stack images were taken using a Nikon confocal microscope. **b** Barplots of cellular volume of three morphotypes

Supplementary material 3 (EPS 7995 kb) Histogram showing the numbers of uniquely mapped reads in all cells

Supplementary material 4 (EPS 1641 kb) Enriched GO terms in β1-integrin-inhibited morphotypic comparisons. **a** Enriched GO terms, comparing β1-integrin-inhibited AR1 vs β1-integrin-inhibited AR7. **b** Enriched GO terms, comparing β1-integrin-inhibited AR11 vs β1-integrin-inhibited AR7. **c** Enriched GO terms, comparing β1-integrin-inhibited AR11 vs β1-integrin-inhibited AR1. GO terms are sorted according to their false discovery rate-adjusted *p* values. “Counts” represents the number of differentially expressed genes in each GO term. “GeneRatio” stands for the ratio of the “Counts” to the total number of genes in each GO term

Supplementary material 5 (EPS 1815 kb) Enriched GO terms in Src-overexpressed morphotypic comparisons. **a** Enriched GO terms, comparing Src-overexpressed AR1 vs Src-overexpressed AR7. **b** Enriched GO terms, comparing Src-overexpressed AR11 vs Src-overexpressed AR7. Src-overexpressed AR11 vs Src-overexpressed AR1 is not presented, since no pathway is significantly enriched in this comparison. GO terms are sorted according to their false discovery rate-adjusted *p* values. “Counts” represents the number of differentially expressed genes in each GO term. “GeneRatio” stands for the ratio of the “Counts” to the total number of genes in each GO term

Supplementary material 6 (XLSX 156 kb)

Supplementary material 7 (XLS 1194 kb)

Supplementary material 8 (XLSX 40 kb)

Supplementary material 9 (XLSX 24 kb)

Supplementary material 10 (XLSX 32 kb)
